# Treatment of Ulnar Coronoid Process Fracture Using the Anterior Neurovascular Interval Approach: A Retrospective Clinical Study with Short‐ to Mid‐term Follow‐up

**DOI:** 10.1111/os.14123

**Published:** 2024-06-03

**Authors:** Fei Yang, Zeyong Wang, Tangbo Yuan, Jian Qin

**Affiliations:** ^1^ Department of Orthopedics, Sir Run Run Hospital Nanjing Medical University Nanjing China

**Keywords:** Anterior approach, Coronoid process, Elbow fracture, Internal fixation

## Abstract

**Objective:**

Numerous surgical techniques for addressing ulnar coronoid process fractures are available; however, a consensus on the optimal approach remains elusive. This study aimed to use the anterior neurovascular interval approach for the surgical management of ulnar coronoid process fractures and to evaluate its clinical outcomes over short‐ to mid‐term follow‐up.

**Methods:**

This retrospective clinical study included 20 patients with ulnar coronoid process fractures who were treated using the anterior neurovascular interval approach between January 2018 and December 2022. Participants comprised 16 males and four females, aged between 20 and 64 years (mean, 34.3 ± 12.44 years). Clinical and radiological evaluations were based on elbow joint range of motion (ROM), Visual analogue scale (VAS), and Mayo elbow performance score (MEPS). A paired *t*‐test was used to compare the pre‐operative and final follow‐up VAS and MEPS scores.

**Results:**

The follow‐up duration for all patients was at least 12 months (average, 12.65 ± 1.60 months). At the final follow‐up, measurements of elbow ROM included a mean extension of 2.85 ± 3.17°, mean flexion of 135 ± 7.25°, mean pronation of 86.4 ± 4.56°, and mean supination of 84.85 ± 5.54°. All participants reached their target MEPS, with an average score of 97.25 ± 4.72 points, and the final mean VAS score was 0.2 ± 0.52 points. The VAS score was significantly lower and MEPS score was higher at the final follow‐up than those before surgery (*p* < 0.05). Throughout the follow‐up period, all the fractures united, and the stability of the affected elbows was satisfactory.

**Conclusion:**

Employing the anterior neurovascular interval approach for open reduction and internal fixation to manage coronoid process fractures effectively facilitates anatomical restoration and robust fixation of ulnar coronoid process fractures.

## Graphical abstract

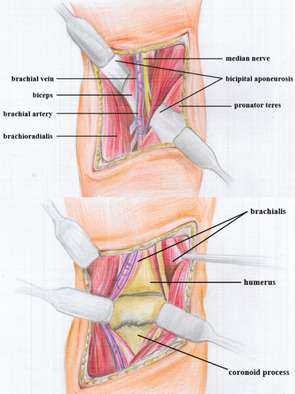

## Introduction

Ulnar coronoid process fractures, which are categorized as intraarticular fractures, are relatively uncommon. These fractures constitute approximately 10% of elbow joint injuries and frequently occur alongside complex injuries and joint dislocations;^1^ the most severe type is the terrible triad, which is usually accompanied by posterior dislocation, radial head fracture, and coronoid process fracture. Approximately 60% of the anterior and medial joints of the ulnar coronoid process lack support from the proximal metaphysis of the ulna, predisposing this area to fractures.^2^ Patients commonly report pain and restricted elbow joint movements. In 1989, Regan and Morrey devised a classification system for ulnar coronoid process fractures, categorizing them as Type I (tip of the coronoid process), Type II (less than 50% of the coronoid process), and Type III (more than 50% of the coronoid process), based on lateral X‐ray images of the elbow joint.^3^ Subsequently, in 2003, O'Driscoll *et al*.^4^ introduced another classification using 3D‐CT reconstructions of the elbow joint, identifying fractures as Type I (tip fracture), Type II (anteromedial fracture), and Type III (basal fracture). Current clinical and biomechanical research suggests that large coronoid process fractures may lead to elbow joint instability and an increased risk of traumatic arthritis.^5^ Consequently, surgical intervention is primarily advocated to restore elbow stability. Prompt post‐injury surgical action and efficient repair of the damaged coronoid process are crucial for early postoperative mobility and rehabilitation of elbow function. However, owing to the unique anatomical structure and complexity of the injury types, the surgical approach and fixation methods for coronoid process fractures remain unclear.

Currently, multiple surgical techniques, including the lateral, posterior, and medial approaches, are employed to treat ulnar coronoid process fractures; however, no approach has been universally accepted. O'Driscoll type II ulnar coronoid process fractures often present with irregular or even comminuted fragments positioned near the anterior or anteromedial side, thus making adequate exposure using lateral or medial approaches challenging. The anterior approach, seldom used for coronoid process fractures, primarily facilitates the exposure and examination of the blood vessels and nerves in the anterior cubital fossa. Reichel *et al*.^6^ described an anterior approach that navigates between the biceps brachii muscle and medial brachial nerve vascular bundle, with the brachial artery and median nerve positioned anteriorly to the elbow joint. A tissue gap that is not tightly packed between the artery and nerve permits entry without damaging the vascular nerve bundle.

This study retrospectively analyzed the clinical data from 20 patients whose ulnar coronoid process fractures were surgically managed with the anterior neurovascular interval approach. This study aimed to: (i) evaluate the clinical outcomes of open reduction and internal fixation through the anterior neurovascular interval approach in the treatment of ulnar coronoid process fractures over short‐ to mid‐term follow‐up periods; (ii) summarize the operational skills and specific precautions for this surgical approach; and (iii) provide reference for the selection of surgical approach for such fractures.

## Materials and Methods

### Inclusion and Exclusion Criteria

Inclusion criteria for this study were: (i) closed ulnar coronoid process fractures without vascular or nerve damage; (ii) diagnosis based on established disease criteria and confirmed using imaging (CT, X‐ray, etc.); (iii) surgical intervention *via* the anterior neurovascular interval approach within 2 weeks post‐injury; and (iv) availability of complete follow‐up data, with a minimum duration of ≥12 months.

Exclusion criteria included: (i) open fractures; (ii) pathological fractures; (iii) history of elbow joint surgery, pre‐existing elbow joint lesions, or movement disorders prior to the injury; (iv) concurrent vascular and neurological injuries; (v) inability to cooperate with treatment or participate in postoperative assessments; (vi) presence of severe physical or mental disorders; and (vii) delay in surgical intervention exceeding 2 weeks post‐injury.

### General Information

This study was approved by the Ethics Committee of our hospital (No. 2023‐SR‐042), and informed consent was obtained from all participants. The cohort comprised 20 patients who underwent open reduction and internal fixation for ulnar coronoid process fractures at our facility between January 2018 and December 2022. The diagnosis was primarily based on clinical findings, complemented by X‐ray and 3D‐CT assessments.

### Surgical Technique

**Figure 1:**
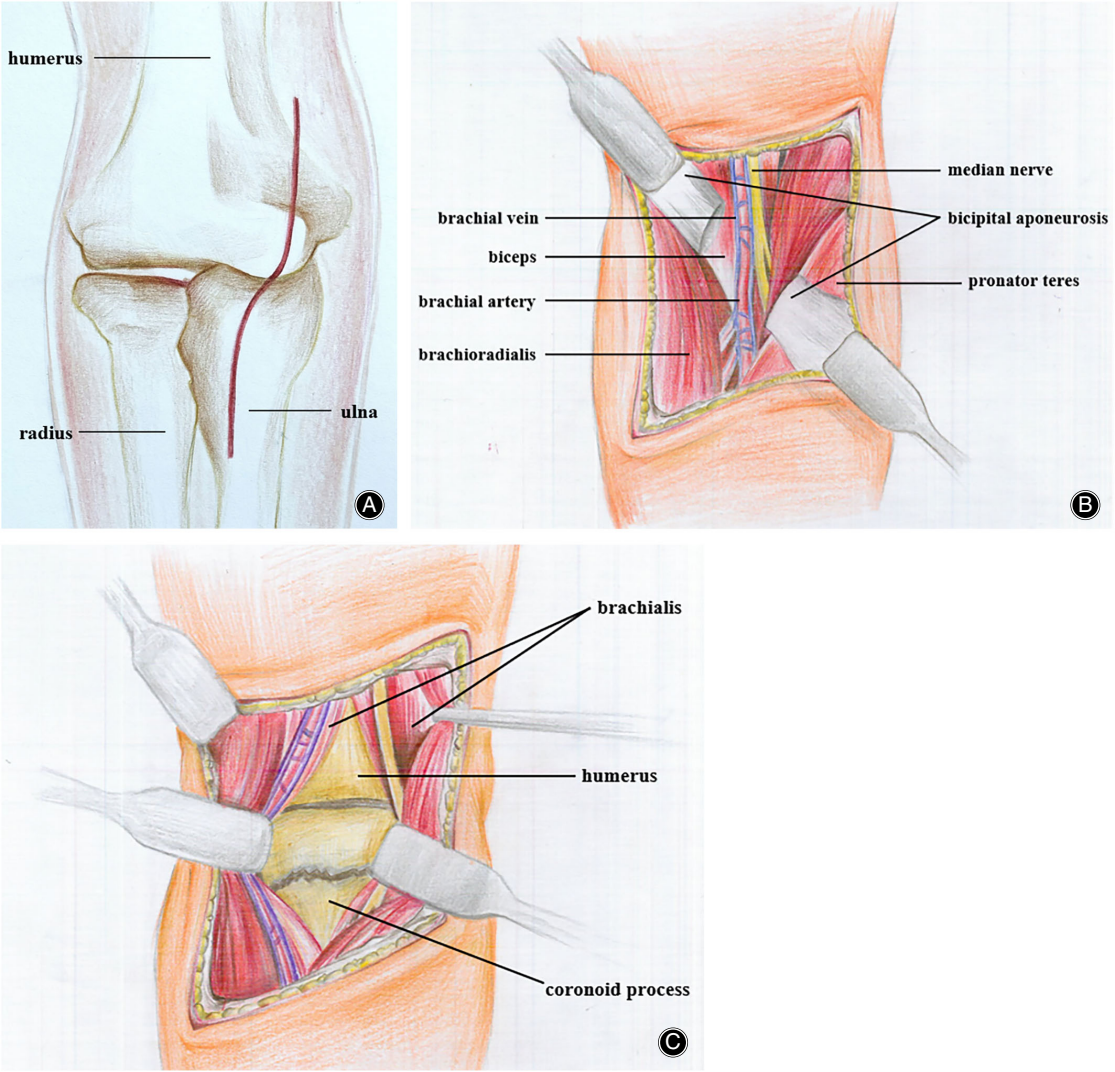
Schematic diagram of the surgical incision and anatomy.

**Figure 2:**
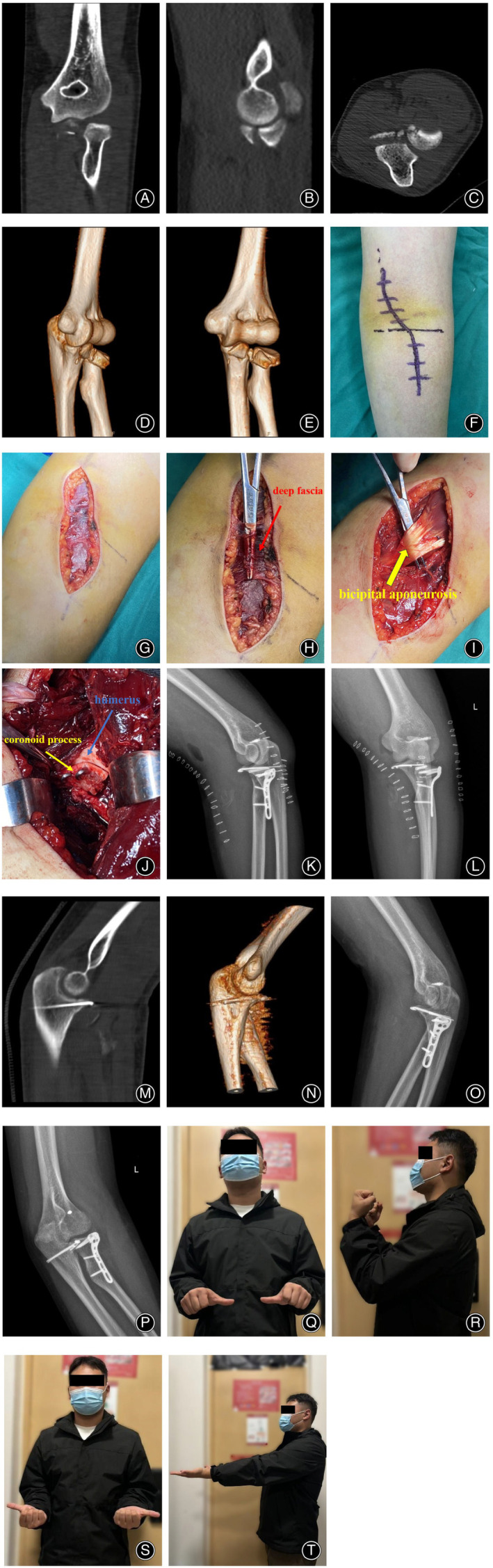
Typical case 1. A 21‐year‐old man suffered from sports‐related injuries and received treatment with k‐wires. (A–E) Preoperative CT images showed ulnar coronoid process fracture and radial head fracture. (F–J) Positioning of the surgical incision and exposure of surgical area. (K–N) Postoperative x‐ray and CT images showed an anatomical reduction of the coronoid process fracture and radial head fracture. (O, P) Ten weeks after the operation, the radiographs of the elbow joint showed that the fracture was completely healed. (Q–T) The function of the patient's elbow joint at the last follow‐up was favorable, and the patient was pain free.

Under general or brachial plexus anesthesia, the patients were positioned supine, and the affected upper limb was routinely disinfected. The surgery was performed using a pneumatic tourniquet. A S‐shaped incision approximately 10 cm in length was created *via* the anterior approach in the cubital fossa (Figures 1A and 2F). The skin, subcutaneous tissue, and deep fascia were sequentially incised (Figure 2G–I), and dissection was performed along the interval between the medial border of the biceps brachii and brachialis, which revealed the brachial artery, brachial vein, and median nerve (Figure 1B). Blunt dissection was performed between the brachial artery and median nerve; followed by lateral retraction of the biceps brachii, brachial artery, and brachial vein; and medial retraction of the median nerve (Figure 1C). The brachialis muscle was longitudinally incised (approximately 6 cm) in line with its fibers to expose the fractured end of the ulnar coronoid process. Fracture reduction was initially achieved by using Kirschner wires. A steel plate was positioned on the coronoid process, followed by sequential drilling, depth measurement, and the insertion of screws of appropriate length (Figure 2J). A C‐arm X‐ray machine was used to ensure accurate reduction and confirm the placement and length of the steel plate and screws. If lateral elbow joint instability was intraoperatively detected, the extensor digitorum communis (EDC) splitting approach was made to repair the compromised collateral ligament. If the ulnar coronoid process fracture is combined with a radial head fracture, internal fixation of the radial head fracture needs to be performed using the EDC splitting approach, and the distance between the two incisions must be greater than 7 cm (Figure 2K,L). The surgery was concluded after checking for active bleeding, irrigation with saline, and incision closure.

### Postoperative Treatment

Routine postoperative measures, such as infection prophylaxis, analgesia, and anti‐inflammatory treatments, were administered. Oral indomethacin enteric‐coated tablets were prescribed for 4 weeks to inhibit heterotopic ossification of the elbow joint. A hinge brace was applied to maintain the affected elbow in a functional position, specifically at a 90° flexion. One week postoperatively, functional exercises, including passive flexion, extension, and rotational movements of the elbow joint, of the limb were commenced under brace protection. After 4 weeks, the brace was removed and active flexion, extension, and rotational exercises were initiated. Weight‐bearing activity was strictly prohibited during this period. Regular imaging follow‐ups with radiography or 3D‐CT at 1, 3, 6, and 12 months postoperatively were scheduled to monitor the fracture healing and guide further functional exercises.

### Follow‐up and Functional Evaluation

Three days postoperatively, radiography and 3D‐CT reconstructions were performed to assess the reduction and fixation results. Subsequent outpatient follow‐ups were scheduled at 1, 3, 6, and 12 months postoperatively with annual checkups thereafter. Dynamic assessments of the fracture healing and internal fixation stability were performed using anterior and lateral radiographs of the elbow joints. At the final follow‐up, elbow joint range of motion (ROM), including flexion, extension, pronation, and supination, was measured. The Visual analogue scale (VAS) and Mayo elbow performance score (MEPS) were used to evaluate elbow joint function.

### Statistical Analysis

All analysis was performed by SPSS version 21.0 software (IBM, Armonk, NY, USA). Normally distributed continuous data are presented as mean ± standard deviation. A paired *t*‐test was used to compare the pre‐operative and final follow‐up VAS and MEPS scores. Statistical significance was set as *p* value <0.05.

## Results

### General Results

The study involved 20 patients, 16 males and four females, aged 20 to 64 years (mean, 34.3 ± 12.44 years), who underwent open reduction and internal fixation of fractures using the anterior neurovascular interval approach, with complete follow‐up data available. Injury causes were predominantly falling (15 patients), followed by traffic incidents (two patients) and sports‐related injuries (three patients). Surgical intervention was more common in the left elbow (*n* = 12) than in the right elbow (*n* = 8). Notably, 12 patients required additional lateral incisions to address the lateral elbow joint instability *via* lateral collateral ligament repair. Fracture classification according to the Regan–Morrey system included three Type I, 15 Type II, and two Type III fractures, while according to O'Driscoll's system, there were two Type I, 16 Type II, and two Type III fractures. The fixation methods varied: 16 patients received mini‐plates, three were treated with Kirschner wires, and one received a combination of both. Surgical durations ranged between 63 and 79 min (mean, 69.05 ± 4.86 min), and perioperative blood loss was reported between 90 and 110 mL (mean, 98.45 ± 5.53 mL) (Table 1).

**TABLE 1:** Patient characteristics.

Patient	Sex	Age	Injury mechanism	Injuried elbow	Regan‐Morrey classification	O'Driscoll classification	Lateral collateral ligament injury	Fixation method	Operation time (min)	Perioperative blood loss (mL)
1	M	21	Sports	Left	II	II	Yes	Kirschner wire	66	90
2	F	22	Fall	Right	II	II	Yes	Mini‐plate	68	96
3	M	46	Fall	Left	I	I	No	Mini‐plate	69	96
4	M	33	Bicycle	Right	II	II	Yes	Mini‐plate	70	105
5	M	28	Fall	Left	II	II	Yes	Kirschner wires	65	98
6	M	48	Fall	Right	II	II	No	Mini‐plate	77	110
7	M	39	Fall	Left	I	II	Yes	Mini‐plate	65	99
8	M	33	Fall	Left	II	II	Yes	Mini‐plate	70	103
9	F	20	Sports	Right	II	I	No	Mini‐plate	68	98
10	F	28	Fall	Left	II	II	No	Mini‐plate and Kirschner wire	79	99
11	M	32	Fall	Left	II	II	No	Mini‐plate	64	90
12	M	37	Fall	Right	II	II	Yes	Mini‐plate	67	93
13	M	44	Fall	Left	II	II	Yes	Mini‐plate	74	104
14	M	30	Fall	Left	II	II	No	Mini‐plate	71	100
15	M	59	Fall	Left	III	III	Yes	Mini‐plate	65	97
16	M	22	Sports	Right	II	II	Yes	Kirschner wire	66	96
17	F	26	Fall	Left	II	II	No	Mini‐plate	75	101
18	M	31	Fall	Right	II	II	No	Mini‐plate	63	93
19	M	64	Fall	Right	II	III	Yes	Mini‐plate	76	108
20	M	23	Bicycle	Left	II	II	Yes	Mini‐plate	63	93

The final outcomes, detailed in Table 2, revealed a minimum follow‐up duration of 12 months for all patients (mean, 12.65 ± 1.60 months; range, 12–18 months). Bony union times varied from 12 to 18 weeks (mean, 13.40 ± 1.43 weeks). Measurements at the final follow‐up showed the following elbow ROM: mean extension 2.85 ± 3.17° (range, 0–10°), mean flexion 135 ± 7.25° (range, 115–145°), mean pronation 86.4 ± 4.56° (range, 75–90°), and mean supination 84.85 ± 5.54° (range, 70–90°). All patients achieved satisfactory MEPS, with a mean score of 97.25 ± 4.72 points (range, 85–100 points), and the final average VAS score was 0.20 ± 0.52 points (range, 0–2 points). The VAS score was significantly lower and MEPS score was higher at the final follow‐up than those before surgery (*p* < 0.05). (Tables 2 and 3).

**TABLE 2:** Outcome details (at final follow‐up).

Patient	Follow‐up time (months)	Union time (weeks)	Extension (°)	Flexion (°)	Pronation (°)	Supination (°)	VAS	MEPS	Complication
1	12	12	0	140	90	90	0	100	None
2	12	13	0	145	88	85	0	100	None
3	12	14	5	130	80	85	0	95	None
4	14	13	4	135	85	85	0	100	None
5	12	13	0	140	90	89	0	100	None
6	12	14	8	125	75	70	1	90	None
7	12	13	4	130	85	85	0	95	None
8	16	13	3	130	90	90	0	100	None
9	12	12	0	145	90	85	0	100	None
10	12	13	0	140	90	88	0	100	None
11	12	14	2	135	85	80	0	100	None
12	13	14	0	135	80	80	0	100	None
13	12	13	7	130	85	80	0	90	None
14	12	13	3	140	90	90	0	100	None
15	18	16	6	130	85	83	1	90	None
16	12	12	0	140	90	90	0	100	None
17	12	13	5	135	90	87	0	100	None
18	12	13	0	140	90	90	0	100	None
19	12	18	10	115	80	75	2	85	None
20	12	12	0	140	90	90	0	100	None

Abbreviations: MEPS: Mayo elbow performance score; VAS, Visual analogue scale.

**TABLE 3:** Comparison of relevant scores between pre‐operation and final follow‐up.

Time	VAS	MEPS
Preoperative	6.70 ± 0.73	47.25 ± 6.38
Last follow‐up	0.20 ± 0.52	97.25 ± 4.72
*t* value	35.14	39.79
*p* value	*p* < 0.05	*p* < 0.05

Abbreviations: MEPS, Mayo elbow performance score; VAS, Visual analogue scale.

### Complications

**Figure 3:**
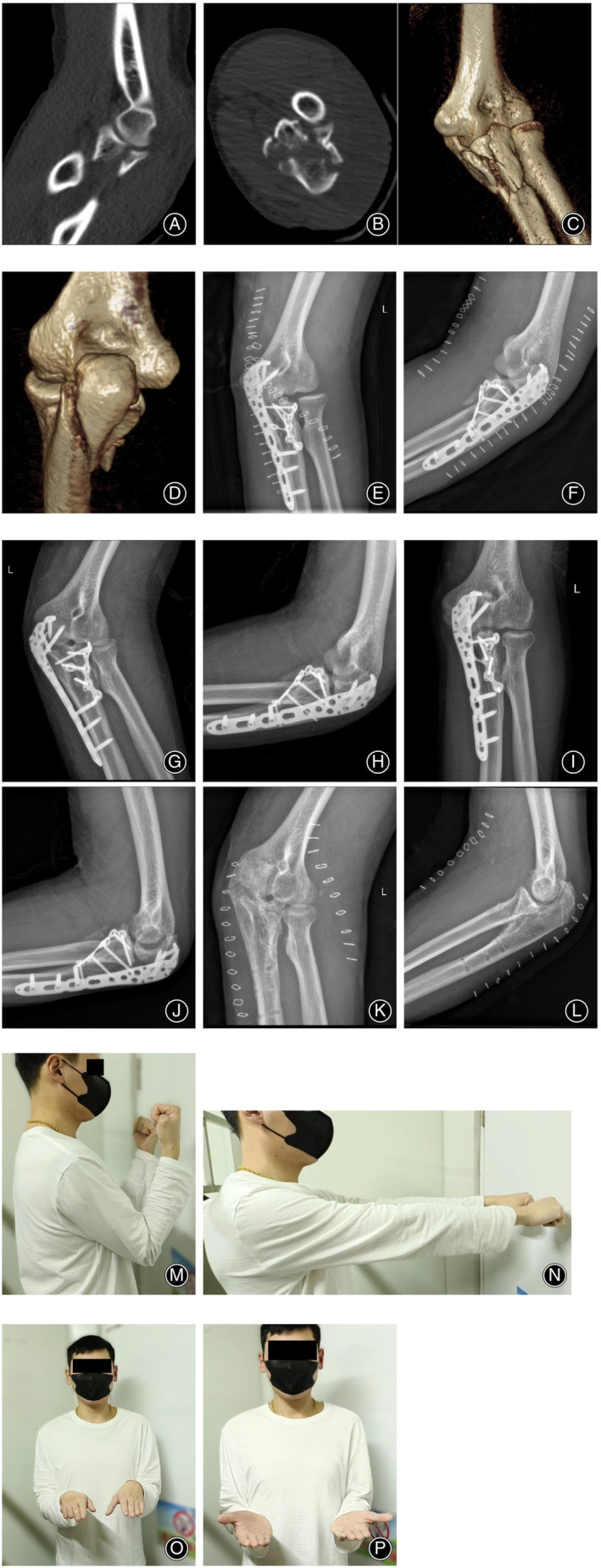
Typical case 2. A 38‐year‐old man suffered from fall‐related injuries. (A–D) Preoperative CT images showed ulnar coronoid process fracture and olecranon fracture. (E, F) Postoperative x‐ray images showed an anatomical reduction of the coronoid process fracture and olecranon fracture. (G, H) Ten weeks after the operation, the radiographs of the elbow joint showed that the fracture was not displaced. (I, J) Seventeen months after the operation, internal fixation was in place and the bone has healed. (K, L) Twenty‐two months after the operation, the internal fixation was removed. (M–P) The function of the patient's elbow joint at the last follow‐up was favorable, and the patient was pain free.

No vascular or nerve injuries were observed during the surgical procedure. Postoperatively, all incisions showed primary healing without infection. Follow‐up assessments confirmed that all fractures were united effectively, and the stability of the treated elbows was satisfactory. Notably, there were no complications, such as internal fixation failure, osteoarthritis, infection, joint incongruency, fracture nonunion, median nerve palsy, or implant failure. The representative cases are shown in Figures 2 and 3.

## Discussion

This study showed favorable clinical and radiographic results by employing the anterior neurovascular interval approach in the surgical management of ulnar coronoid process fractures monitored over an average follow‐up period of 13 months. Among the 20 participants, none encountered intraoperative complications, such as vascular or nerve damage, nor were any postoperative complications reported. At the final follow‐up, elbow's ROM was deemed satisfactory, and both the mean MEPS scores was notably high and VAS scores was markedly low, thus indicating excellent outcomes.

### Coronoid Process is Important for Elbow Stability

The anatomical complexity of the elbow joint is significant. It consists of the distal end of the humerus and the proximal ends of the radius and ulna, forming three articulations: the humeroulnar, humeroradial, and proximal radioulnar joints. Heim *et al*.^7^ introduced the four‐column theory to explore the elbow joint stability by segmenting it into anterior, posterior, medial, and lateral columns. The anterior column comprises the coronoid process, anterior elbow joint capsule, and the brachialis muscle. The posterior column includes the olecranon, posterior elbow joint capsule, and triceps brachii muscle. Medially, the column is formed by the medial humeral epicondyle, coronoid process, and ulnar collateral ligament, whereas laterally, the column consists of the lateral humeral epicondyle, radial head, and radial collateral ligament. The coronoid process, integral to both the anterior and medial columns, is pivotal for elbow joint stability. Projecting forward and upward from the ulnar semilunar notch, the coronoid process is a key component of the humeroulnar joint. It serves as the attachment site for the joint capsule, anterior bundle of the medial collateral ligament, and brachialis muscle. Functionally, the coronoid process not only restricts anterior elbow joint displacement but also substantially contributes to stabilizing the elbow during varus, valgus, and forearm rotational movements. Biomechanical studies have revealed that the posterior axial stability remains largely unaffected in coronoid process fractures, affecting less than 50% of the coronoid plane, provided that there is no severe damage to other auxiliary elbow joint structures. Conversely, compared with intact elbows, fractures encompassing more than 50% of the coronoid plane demonstrate notable posterior axial displacement.^8^, ^9^ Moreover, the coronoid process stabilizes the elbow against varus and valgus stresses, preventing posteromedial or posterolateral rotational instability, respectively.^8^

### Surgical Indications

Coronoid process fractures, which are categorized as intraarticular fractures, frequently co‐occur with anterior capsule and ligament ruptures, radial fractures, and olecranon fractures. Larger bone fragments often induce joint instability, whereas smaller fragments have less impact on elbow joint stability. Smaller bone fragments may be more amenable to conservative treatment.^10^ Garrigues *et al*.^11^ posited that irrespective of the ulnar coronoid process fracture type, the reliance on external fixation devices and the necessity for medial collateral ligament repair could be markedly reduced with diverse reduction techniques and stable fixation of the fracture segment. Chemama *et al*.^12^ advocated the prompt and effective reduction of small coronoid process fractures to ensure the early restoration of optimal elbow joint function. For Regan–Morrey and O'Driscoll Type I coronoid process fractures that cannot be fixed with steel plates or Kirschner wires, plasters or braces are usually used to fix and immobilize the injured elbow joint, if the fracture is not displaced or has a small displacement. The fixation time depends on the healing of the fracture, which is usually 1 month. If there is a large displacement, we usually use the suture lasso technology for fixation. In this study, we performed surgical treatment regardless of the size of the bone fragment in coronoid process fractures.

### Selection of Operative Approach for Coronoid Process Fractures

A standardized surgical protocol for the fixation of ulnar coronoid process fractures remains elusive. Treatment should adhere to intraarticular fracture management principles, emphasizing anatomical restoration of the articular surface, reinstatement of elbow joint stability, and initiation of early elbow joint functional exercises. Inadequate management of coronoid process fractures may result in severe complications, such as habitual joint dislocation, stiffness, limited flexion/extension, and traumatic osteoarthritis.

Determining optimal surgical exposure while minimizing intraoperative trauma is fundamental for successful surgery. Current surgical approaches include medial, lateral, and posterior approaches.

The medial approach is optimal for managing medial collateral ligament injuries, noncomminuted large and relatively intact coronoid process fractures, and anteromedial facet fractures of the coronoid process.^13^, ^14^ Prominent medial approaches include the flexor carpi ulnaris‐splitting and Hotchkiss over‐the‐top approaches. The flexor carpi ulnaris‐splitting approach is widely used for its extensive exposure of the anterior medial aspect of the ulnar coronoid process, proximal ulna, and medial collateral ligament, and offers an exposure area approximately three times larger than that of the Hotchkiss over‐the‐top approach. However, it involves extensive soft tissue dissection and long incisions, potentially risking damage to the common flexor tendon, ulnar collateral ligament, and ulnar nerve during surgery.^2^ Additionally, the medial approach may necessitate humeral medial epicondyle osteotomy to enhance coronoid process exposure, and achieving vertical plate fixation can be challenging.^15^

The lateral approach is primarily indicated for injuries to the lateral structures of the elbow joint, such as fractures of the radial head, humeral lateral condyle, and lateral collateral ligament. However, direct exposure of the coronoid process is limited, rendering it suboptimal for isolated coronoid process fractures.^15^, ^16^ This approach involves stripping the brachialis and anterior joint capsules to visualize the coronoid process without necessitating radial head removal. However, achieving precise reduction and robust fixation of anteromedial ulnar coronoid process fractures are challenging. During coronoid process exposure *via* the lateral approach, a (45.4 ± 4.8) mm incision through the extensor digitorum communis muscle is required, posing risks to the posterior interosseous nerve. Furthermore, intraoperative liberation of the lateral collateral ligament may compromise posterolateral rotational stability of the elbow joint.^17^ The lateral approach entails more extensive soft tissue dissection and greater trauma than medial approach, thereby potentially leading to postoperative complications, such as myositis ossificans and elbow stiffness.

In this study, we used the EDC splitting approach, when coronoid process fracture is combined with radial head fracture and/or lateral collateral ligament injury, for reduction of the radial head fracture and repair of the lateral collateral ligament. The EDC splitting approach starts from the lateral epicondyle of the humerus and extends longitudinally along the middle of the extensor digitorum communis muscle to the distal forearm. EDC splitting approach is suitable for radial head fixation, radial head replacement, and lateral collateral ligament complex repair, and can simultaneously handle O'Driscoll Type I and Type II ulnar coronoid process fractures that do not require plate internal fixation. It can fully expose the anterior medial radial head, reduce soft tissue detachment, and lower the probability of iatrogenic lateral ulnar collateral ligament injury. Desloges *et al*.^18^ established the three‐dimensional model based on data from 10 upper limb specimens and found that the EDC splitting approach could expose 100% of the anterior aspect of the radial head and approximately 23% of the height of the coronoid process. But with the radial nerve passing through the distal end of the EDC splitting approach, the exposure range toward the distal end should be controlled within 4 cm of the distal end of the humeroradial joint. During our surgery, the distance between the two incisions was kept greater than 7 cm to prevent skin flap necrosis.

The posterior approach poses challenges in exposing and securing the coronoid process without olecranon resection, making it primarily applicable in cases of concurrent coronoid process and olecranon fractures. This approach involves a posterior median incision of the elbow joint, removal of the olecranon fracture segment for anterior coronoid process exposure, followed by reduction and fixation of the coronoid process, and ultimately, olecranon restoration.^15^ Although the posterior approach minimizes the risk of cutaneous nerve damage, it necessitates long incisions and extensive soft tissue dissection, thereby increasing the chances of complications, such as hematoma formation and skin necrosis.^19^

### Advantages, Disadvantages, and Surgical Techniques of the Anterior Neurovascular Interval Approach

The anterior neurovascular interval approach consists of dissecting loose tissue between the brachial artery and median nerve and pulling the brachial artery toward the radial side and the median nerve toward the ulnar side to reveal the brachialis. Subsequently, the brachialis was split along its fibers to expose the joint capsule. Once the joint capsule is incised, the surgeon gains direct access to the coronoid process fracture fragments and the joint surface for reduction and fixation. The merits of this approach are as follows: (i) the anatomical landmarks are distinct and superficial, facilitating easy access between the brachial artery and median nerve; (ii) it preserves the elbow joint's normal anatomy, safeguarding structures, such as the medial collateral ligament, flexor muscles, pronators, and ulnar nerve from damage; (iii) after blunt dissection, minimal force is needed to pull the brachial artery toward the radial side and the median nerve toward the ulnar side, offering a comprehensive view of the surgical area while easing the tension on the brachial artery, vein, and median nerve; (iv) this approach allows for reduction under direct vision, enabling the placement of screws perpendicularly to the fracture line from anterior to posterior for effective compression fixation of the fracture segment and anatomical realignment of the ulnar coronoid process; (v) this approach aids in the repair of the joint capsule, thus enhancing the stability of the elbow joint; and (vi) it minimizes extensive soft tissue dissection, thereby lowering the risk of complications, such as myositis ossificans and elbow stiffness. Although the approach through the vascular nerve gap grants ample exposure to the apex of the coronoid process and allows the identification of articular cartilage injuries within the joint by flexing the elbow, access to the inner side is somewhat restricted. The variability in the median nerve anatomy at the elbow indicates that this approach is not universally applicable, with adequate dissection unattainable in all patients. After complete dissection, the capacity of the vascular nerves to endure traction is limited, particularly for the nerves. This constraint inherently restricts the surgical workspace and increases the risk of iatrogenic injury due to traction applied to the nerves and blood vessels. Furthermore, this method offers limited exposure to the anterior bundle of the medial collateral ligament, posing challenges in managing complex fractures.^13^ In this study, postoperative nerve traction symptoms were notably absent, possibly because of the extensive branching of the median nerve into the superficial flexor muscle, palmaris longus, and flexor carpi radialis, which permits muscle traction without inflicting nerve damage. This study also highlighted the advantages of the interval approach in reducing intraoperative bleeding, shortening the surgical duration, and significantly reducing damage to the elbow joint.

The anterior neurovascular interval approach requires competent operational skills and adherence to specific precautions to ensure success and minimize risks: (i) the elbow should be flexed to 30–45° to optimize soft tissue traction and enhance fracture exposure; (ii) when longitudinally splitting the brachialis muscle, it is crucial to gently retract the edges of the split muscle to prevent potential damage to nearby blood vessels and nerves; (iii) care should be taken for the lateral cutaneous nerve of the forearm, which runs along the radial side of the brachialis at the elbow, and gentle manipulation of the brachialis is essential to avoid nerve damage; (iv) during joint capsule repair, positioning the joint in extension is advised to prevent overly tight sutures, which can complicate elbow extension postoperatively because of contracture or scar hyperplasia of the anterior joint capsule; (v) clear exposure of the median nerve, brachial artery, and vein is paramount during surgery. Although navigating the space between these structures may seem straightforward, there is a persistent risk of damaging vessels and nerves during operative maneuvers within these gaps; and (vi) elevation of the affected limb for 3–5 mins, foregoing a blood‐repellent belt, can facilitate venous congestion. This technique simplified the identification of blood vessel pathways during surgery, thereby lowering the likelihood of vascular injuries.

### Limitations

This study has some limitations. The incidence of isolated ulnar coronoid process fractures is relatively low and extensive comparative clinical studies on various surgical approaches and treatment methodologies are currently lacking. Owing to the limited number of cases, a statistical analysis could not be performed in this study. In addition, the ROM and functional scores of the affected elbow joint are affected by radial head fractures and injuries to the medial and lateral ligaments. Finally, the study was constrained by its relatively short follow‐up duration, highlighting the need for medium‐ to long‐term follow‐up studies to thoroughly assess efficacy. In the future, we will continue to perform long‐term follow‐up studies to further evaluate the long‐term effects and prognosis of the anterior neurovascular interval approach and to promote continuous innovation and progress in this surgical approach.

## Conclusions

The findings of this study highlight the effectiveness of the anterior neurovascular interval approach in providing substantial exposure for ulnar coronoid fractures. This approach proved advantageous in achieving anatomical reduction and robust fixation of fractures. Characterized by its straightforward procedure, high efficiency, and low complication rate, the anterior neurovascular interval approach shows promising short‐term outcomes. This is a viable surgical option for treating ulnar coronoid fractures.

## Author Contributions

Jian Qin and Tangbo Yuan designed the study. Tangbo Yuan operated on the patients and performed the postoperative follow‐up. Fei Yang and Zeyong Wang collected and analyzed the data and prepared the manuscript.

## Funding Information

This study was supported by the Nanjing Health Science and Technology Development Special Fund Project (YKK22207) and Medical research projects of Jiangsu Provincial Health Commission in 2023 (K2023048, Z2023085).

## Conflict of interest Statement

The authors declared no potential conflicts of interest with respect to the research, authorship, and/or publication of this article.

## Ethics Statement

This study was approved by the Ethics Committee of Sir Run Run Hospital, Nanjing Medical University (approval number: No. 2023‐SR‐042), and all methods were performed in accordance with the Declaration of Helsinki and other relevant guidelines and regulations. Informed consent was obtained from all individual participants included in the study.

## References

[1] Labronici PJ , Belangero WD , Zublin CM , Gonçalves LBJ , Fajardo H , Pires RE , et al. Morphological characteristics of proximal ulna fractures: a proposal for a new classification and agreement for validation. Healthcare. 2023;11(5):693. 36900697 10.3390/healthcare11050693 PMC10000609

[2] Shen JJ , Qiu QM , Gao YB , Tong SL , Huang JF . Direct anterior approach for mini plate fixation of Regan‐Morrey type II comminuted ulnar coronoid process fracture. J Orthop Surg. 2019;27(1):2309499018825223. 10.1177/2309499018825223 30798735

[3] Regan W , Morrey B . Fractures of the coronoid process of the ulna. J Bone Joint Surg Am. 1989;71(9):1348–1354. 2793888

[4] O'Driscoll SW , Jupiter JB , Cohen MS , Ring D , McKee M . Difficult elbow fractures: pearls and pitfalls. Instr Course Lect. 2003;52:113–134. 12690844

[5] Zhang HL , Lin KJ , Lu Y . Prediction of the size of the fragment in comminuted coronoid fracture using the contralateral side: an analysis of similarity of bilateral ulnar coronoid morphology. Orthop Surg. 2020;12(5):1495–1502. 33017086 10.1111/os.12780 PMC7670165

[6] Reichel LM , Milam GS , Reitman CA . Anterior approach for operative fixation of coronoid fractures in complex elbow instability. Tech Hand Up Extrem Surg. 2012;16(2):98–104. 22627936 10.1097/BTH.0b013e31824e6a74

[7] Heim U , Bühler M . Kombinierte verletzungen von radius und ulna im proximalen unterarmsegment. In: Holz U , Rehm KE , editors. 57. Jahrestagung der Deutschen Gesellschaft für Unfallchirurgie e.V. Berlin Heidelberg GmbH: Springer‐Verlag; 1994. p. 61–79.

[8] Rausch V , Jettkant B , Lotzien S , Rosteius T , Mempel E , Schildhauer TA , et al. Biomechanical comparison of screw osteosyntheses and anatomical plating for coronoid shear fractures of the ulna. Arch Orthop Trauma Surg. 2021;141(9):1509–1515. 33044707 10.1007/s00402-020-03621-1 PMC8354969

[9] Zhao S , Zeng C , Yuan S , Li R . Reconstruction of coronoid process of the ulna: a literature review. J Int Med Res. 2021;49(4):3000605211008323. 33858252 10.1177/03000605211008323 PMC8053771

[10] Jo SW , Shin DJ . The novel hooked Kirschner wire technique for ulna coronoid process fractures. Clin Orthop Surg. 2023;15(1):127–134. 36778994 10.4055/cios22148 PMC9880501

[11] Garrigues GE , Wray WH 3rd , Lindenhovius AL , et al. Fixation of the coronoid process in elbow fracture‐dislocations. J Bone Joint Surg Am. 2011;93(20):1873–1881. 22012524 10.2106/JBJS.I.01673

[12] Chemama B , Bonnevialle N , Peter O , Mansat P , Bonnevialle P . Terrible triad injury of the elbow: how to improve outcomes? Orthop Traumatol Surg Res. 2010;96(2):147–154. 20417913 10.1016/j.rcot.2010.02.008

[13] Aggarwal S , Paknikar K , Sinha J , Compson J , Reichert I . Comprehensive review of surgical approaches to the elbow. J Clin Orthop Trauma. 2021;20:101482. 34262848 10.1016/j.jcot.2021.101482 PMC8254122

[14] Ni Q , Yang X , Pan Z , Wang J . The pronator teres and the flexor carpi radialis interval approach for operative fixation of ulna coronoid process fractures. Orthop Traumatol Surg Res. 2021;107(2):102610. 32418740 10.1016/j.otsr.2020.04.004

[15] Orbay JL , Heifner JJ , Gray RRL , Rubio F , Hoekzema NA , Mercer DM . A medial approach that provides ample exposure of the coronoid for fracture management. Tech Hand Up Extrem Surg. 2023;27(4):214–219. 37439145 10.1097/BTH.0000000000000444 PMC10651265

[16] Yang XH , Wei C , Li GP , Wang JJ , Zhao HT , Shi LT , et al. Anatomical study of the anterior neurovascular interval approach to the elbow: observation of the neurovascular interval and relevant branches. Folia Morphol. 2020;79(2):387–394. 10.5603/FM.a2019.0093 31448401

[17] Sukegawa K , Suzuki T , Ogawa Y , Ueno K , Kiuchi H , Kanazuka A , et al. Anatomic cadaveric study of the extensile extensor digitorum communis splitting approach for exposing the ulnar coronoid process. J Shoulder Elbow Surg. 2016;25(8):1268–1273. 27032618 10.1016/j.jse.2016.01.008

[18] Desloges W , Louati H , Papp SR , Pollock JW . Objective analysis of lateral elbow exposure with the extensor digitorum communis split compared with the Kocher interval. J Bone Joint Surg Am. 2014;96(5):387–393. 24599200 10.2106/JBJS.M.00001

[19] Li D , Song D , Ni J , Tang S , Gao Z , Li P , et al. Single modified posterior approach through the space of the proximal radioulnar joint for terrible triad injury: a comparative study. Orthop Surg. 2022;14(9):2159–2169. 35929666 10.1111/os.13430 PMC9483065

